# Minimally invasive swine spine surgery training: technical aspects, benefits, and anatomical limitations

**DOI:** 10.31744/einstein_journal/2022AO6318

**Published:** 2022-02-02

**Authors:** Alberto Ofenhejm Gotfryd, Fábio Chaud de Paula, Marcel Lobato Sauma, Alexandre Sadao Iutaka, Luciano Miller Reis Rodrigues, Guilherme Pereira Correa Meyer, Marcelo Passos Teivelis, Arthur Werner Poetscher, David Del Curto, Davi Wen Wei Kang, Luciana Cintra, Guilherme Buzon Gregores, Mario Lenza, Mario Ferretti

**Affiliations:** 1 Hospital Israelita Albert Einstein São Paulo SP Brazil Hospital Israelita Albert Einstein, São Paulo, SP, Brazil.; 2 Escola Paulista de Medicina Universidade Federal de São Paulo São Paulo SP Brazil Escola Paulista de Medicina, Universidade Federal de São Paulo, São Paulo, SP, Brazil.

**Keywords:** Simulation training, Models, animal, Orthopedic procedures, Microsurgery, Spine/surgery, Decompression, surgical, Minimally invasive surgical procedures, Swine

## Abstract

**Objective:**

To describe the technical specificities and feasibility of simulation of minimally invasive spine surgery in live pigs, as well as similarities and differences in comparison to surgery in humans.

**Methods:**

A total of 22 Large White class swine models, weighing between 60 and 80kg, were submitted to surgical simulations, performed during theoretical-practical courses for training surgical techniques (microsurgical and endoscopic lumbar decompression; percutaneous pedicular instrumentation; lateral access to the thoracic spine, and anterior and retroperitoneal to the lumbar spine, and management of complications) by 86 spine surgeons. For each surgical technique, porcine anatomy (similarities and differences in relation to human anatomy), access route, and dimensions of the instruments and implants used were evaluated. Thus, the authors describe the feasibility of each operative simulation, as well as suggestions to optimize training. Study results are descriptive, with figures and drawings.

**Results:**

Neural decompression surgeries (microsurgeries and endoscopic) and pedicular instrumentation presented higher similarities to surgery on humans. On the other hand, intradiscal procedures had limitations due to the narrow disc space in swines. We were able to simulate situations of surgical trauma in surgical complication scenarios, such as cerebrospinal fluid fistulas and excessive bleeding, with comparable realism to surgery on humans.

**Conclusion:**

A porcine model for simulation of minimally invasive spinal surgical techniques had similarities with surgery on humans, and is therefore feasible for surgeon training.

## INTRODUCTION

The growing demand for surgical techniques in recent decades has stimulated the emergence of new methods for teaching and training surgeons.^(1)^ Minimally invasive surgeries for the spine stand out, and despite their advantages over conventional techniques,^(2)^ they are related to longer learning curves.^(3,4)^ Moreover, modern techniques require technological resources, which are not always available in traditional teaching centers.

Among the types of simulation, using live animals is the closest to real life.^(5,6)^ The main advantages include hemorrhage control and handling of tissues with consistency similar to human ones, in addition to encouraging teamwork and the division of responsibilities in the operating field.^(7)^

Due to the similarity in anatomy, pigs are used for training surgeons in several medical fields. As far as the authors are aware, there has been no description in the literature of training for minimally invasive spinal procedures in swine. The authors hypothesize that it is possible to train such surgical techniques in an experimental swine model, achieving realism similar to that observed in surgeries performed on humans.

## OBJECTIVE

To describe the technical specificities and feasibility of simulations of minimally invasive spine surgery in live pigs, as well as similarities and differences in comparison to surgery on humans.

## METHODS

This article is an experimental study, developed at *Hospital Israelita Albert Einstein*. The present study was approved by the Ethics Committee on Animal Use (CEUA/Einstein) of the organization, *as per* opinions – CEUA 2367/2015, CEUA 2781/2016, CEUA 3193/2017, CEUA 3214/2017 and CEUA 3568-18 –, and was performed at the Animal Facility, accredited by the Association for Assessment and Accreditation of Laboratory Animal Care International (AAALAC International).

Between April 2015 and October 2017, 22 pigs were submitted to surgical simulations carried out during ten theoretical-practical courses for training spine surgeons. Each event addressed different minimally invasive spinal techniques. Students were divided into practice stations and supervised by two senior surgeons with proven experience in the technique in humans.

Animals were Large White class, aged 7 months and weighing between 60 and 80kg. Prior to the anesthetic procedure, the animals were sedated with ketamine (10mg/kg) and midazolam (0.25mg/kg). Anesthesia induction was performed with propofol (5mg/kg), followed by orotracheal intubation, and were maintained with 2% isoflurane. Mechanical ventilation with a mixed volume of 1L of compressed air and respiratory volume of 8mL/kg was used. Analgesia was performed with tramadol (2mg/kg) and continuous infusion of fentanyl (2mL/hour).

Endoscopic surgery of the transforaminal and interlaminar lumbar spine, over-the-top lumbar decompression, split lumbar decompression of the spinous process, percutaneous pedicle instrumentation, lateral access to the thoracic spine (minithoracotomy) and retroperitoneal anterior access to the lumbar spine were the techniques performed, and complications such as durotomy and bleeding were managed.

At the end of each training session, a consensus meeting was held between senior surgeons and students to discuss the feasibility of surgical techniques in pigs, in addition to comparing them with surgery in humans. For each technique assessed, the following topics were considered: animal positioning; access route and anatomical repairs; tissue consistency and appearance; bone anatomy; neural and vascular anatomy; possibility of instrumentation and size of implants. For educational purposes, at the end of each technique described in this study, strengths and weaknesses wee pointed out, according to the similarities or differences in relation to surgeries performed in humans.

Study results are descriptive (step-by-step surgery) and shown by drawings, photographs, and radioscopy images.

## RESULTS

### Positioning

**Figure 1:**
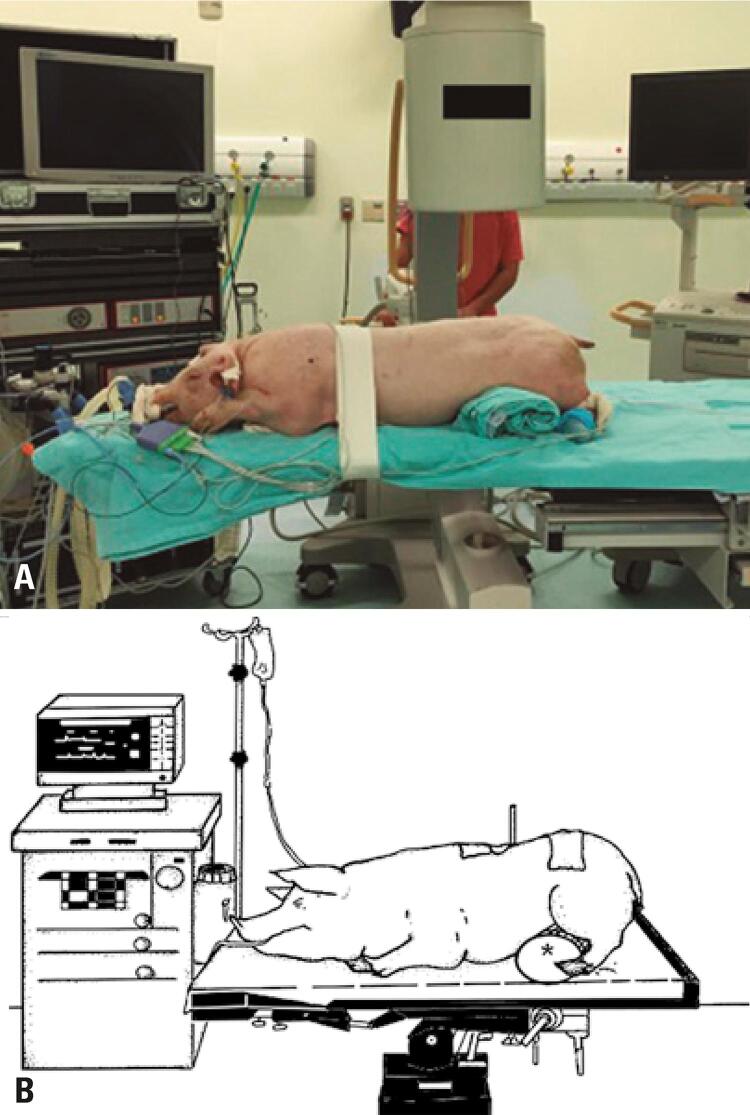
(A and B) Positioning of the animal for posterior access route with support under the lower limb to reduce abdominal pressure

For surgical techniques with a dorsal approach, the animal was positioned in prone, with a pad located anteriorly to the hind legs, to reduce abdominal pressure and, consequently, intraoperative bleeding (Figures 1A and 1B). In the minithoracotomy technique, the animal was positioned in lateral decubitus (contralateral to the one approached), and in the lumbar retroperitoneal approach, positioning was horizontal dorsal decubitus.

### Transforaminal lumbar endoscopic approach technique

The interlaminar and transforaminal Karl Storz Thoracic and Lumbar Percutaneous Endoscopic System (Spine TIP^®^ -Germany) was used for endoscopic surgeries.

**Figure 2:**
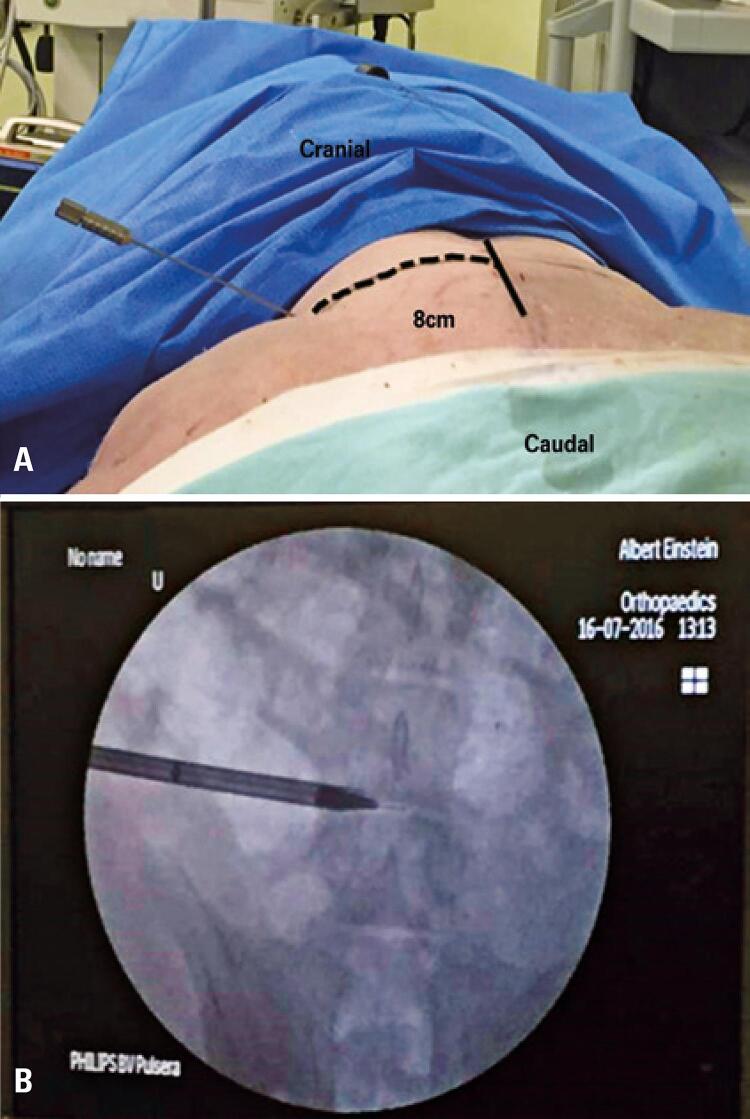
(A) Needle positioning for initial access of transforaminal endoscopy; (B) Radioscopic image of the initial dilator for transforaminal endoscopic access

The procedure started by placement of a needle, which was introduced in the posterolateral lumbar region, about 8cm from the midline to the disc, guided by radioscopy (Figures 2A and 2B). A path with an angle between 10° and 20° in relation to the posterior portion of the disc was used, similar to the one used for humans.

The needle reached the disc in a triangle formed by the emerging root, dura mater, and upper portion of the inferior vertebral pedicle (Kambin’s safety triangle).^(8)^ The position of the needle was checked in anteroposterior and lateral orthogonal views, for the disc to be penetrated into this triangle.

A guidewire was passed through the needle, then removed, and a 6.9mm diameter dilator was introduced into the disc space. Then, an 8mm outer diameter cannula was introduced over the dilator, and its position was checked by radioscopy images.

A hammer was used to pass the dilator, and later, a cannula. The dilator deformed the endplates when entering the animal’s disc space, but this did not prevent passing the jacket, and later, the endoscope.

Once the instruments were properly positioned, the visualization of the epidural space, nerve roots and dura mater was similar to that of a human.

In this technique, the reduced height of the lumbar conjugation foramen was considered as a divergent point from surgery in humans (even at lower anatomical levels). Thus, when positioning the working cannula, deformation of the end plate occurred sometimes, but it did not affect the quality of the endoscopic image obtained. In addition, the reduced disc height made it difficult to enter and remove disc content.

### Interlaminar endoscopic lumbar approach technique

Similar to what is found in humans, the dimensions of the interlaminar window in the pig were larger in the more distal portions, especially in the lumbosacral transition, and progressively decreased at the more cranial levels. This characteristic imposed more difficulties in accessing the vertebral canal in the more proximal segments and was due to the reduction in the distance between the facets, which narrowed the window and deepened the ligamentum flavum. On the other hand, it reproduced, more reliably, the situation found in canal stenosis, and was able to be a model for training more advanced techniques to more experienced surgeons.

The procedure started with locating the interlaminar window by anteroposterior radioscopy. The articular facet and the ligamentum flavum with an initial cannula were identified, and the depth was verified by means of lateral radioscopy.

Then, the ligamentum flavum was opened with endoscopic scissors. The ligamentum flavum was thinner in the pig, but had a consistency similar to that of a live human. Thus, entry into the animal’s spinal canal was easier, and the fat and epidural vessels could be visualized right away, covering the dural sac and descending roots, in the same way as observed in humans.

Navigation inside the canal was performed with optics and mobilization of neural elements. The anatomical distribution of the dural sac and descending roots within the vertebral canal was also similar to that of humans. Thus, neural tissue manipulation maneuvers to expose the disc were possible by using the spatula and the rotation of the working cannula bevel.

The strengths were the interlaminar space anatomy similar to that of humans and the possibility of replicating the technique at multiple lumbar levels. The weak point was the fact that a rudimentary intervertebral disc made it difficult to perform both interlaminar and transforaminal discectomy.

### Tubular neural lumbar decompression technique

An OPMI Pentero microscope (Carl Zeiss Meditec AG, Germany), Metrx tubular retractors (Medtronic Sofamor Danek Usa, Inc), pneumatic drill (Midas Rex, Medtronic Sofamor Danek) and fluoroscopy were used for all microsurgical decompression surgeries.

### Over-the-top decompression

The technique was possible on at least five lumbar levels. Anatomical bone repairs, such as the interlaminar space and neural structures (dural sac and root distribution), were similar to humans.

The anatomical level was checked by profile fluoroscopic imaging. The height of the disc space and the midline were marked. Since the porcine paraspinal muscle attachment is exuberant, the skin incision was made no more than 5mm away from the midline, and was slightly more extensive (3cm) than that made in humans.

After positioning the soft tissue retractor, a laminotomy was performed using a drill. At this stage, care was required so as to not injure the ligamentum flavum by using an electric cutter, given the ligamentum flavum is thinner and less resistant in pigs.

**Figure 3:**
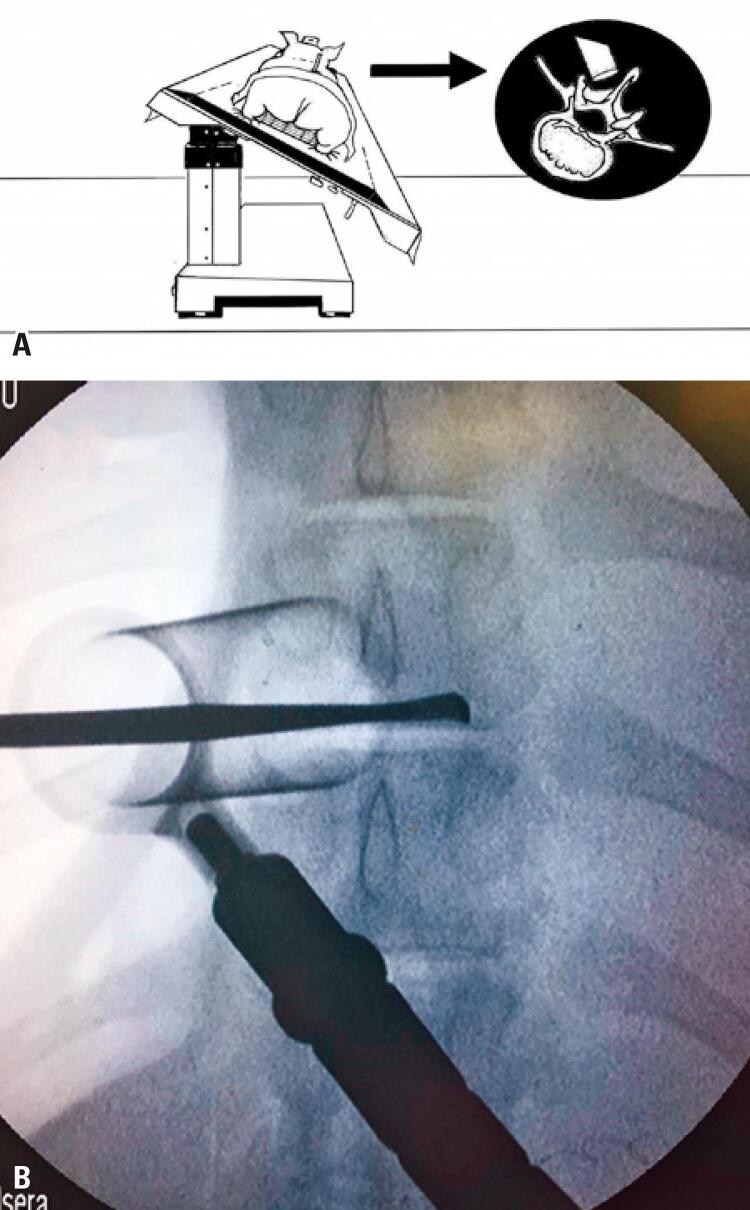
(A) Table rotation for over-the-top access; (B) Radioscopic image of over-the-top decompression. The Penfield is positioned through the median line, in the pedicular region of the contralateral side

After the ipsilateral laminotomy, the surgical table was tilted contralaterally (roughly 30°), and the tube was tilted ipsilaterally, so as to obtain a tunnel view in the contralateral direction (Figures 3A and 3B).

The base of the spinous process was resected using the drill. The porcine spinous process proved to be longer than in humans. The resection of the most ventral portion of the spinous process, adjacent to the ligamentum flavum, was sufficient to visualize the contralateral lamina.

A contralatreal laminotomy was then performed. The swine spine is narrower than the human spine, so little resection of the contralateral lamina was required to visualize the contralateral edge of the dura mater, ending the training.

The strong point was the similarity of the vertebral canal anatomy in pigs and humans. The weak point was the exuberant porcine dorsal musculature, which made it difficult to position the tubular retractor close to the midline.

### Lumbar spinous process splitting decompression

For this technique, the main difference found was a deeper osteotomy than what is performed in humans, due to the greater length of the swine spinous process.

The procedure began with a midline incision (3cm) between the superior and inferior spinous processes of the one intended for access. The spinous process was divided longitudinally using a diamond bur, with the paravertebral muscles remaining attached to its sides. The supraspinatus and interspinous ligaments were incised and divided longitudinally. The musculature inserted in the blade was dissected.

**Figure 4:**
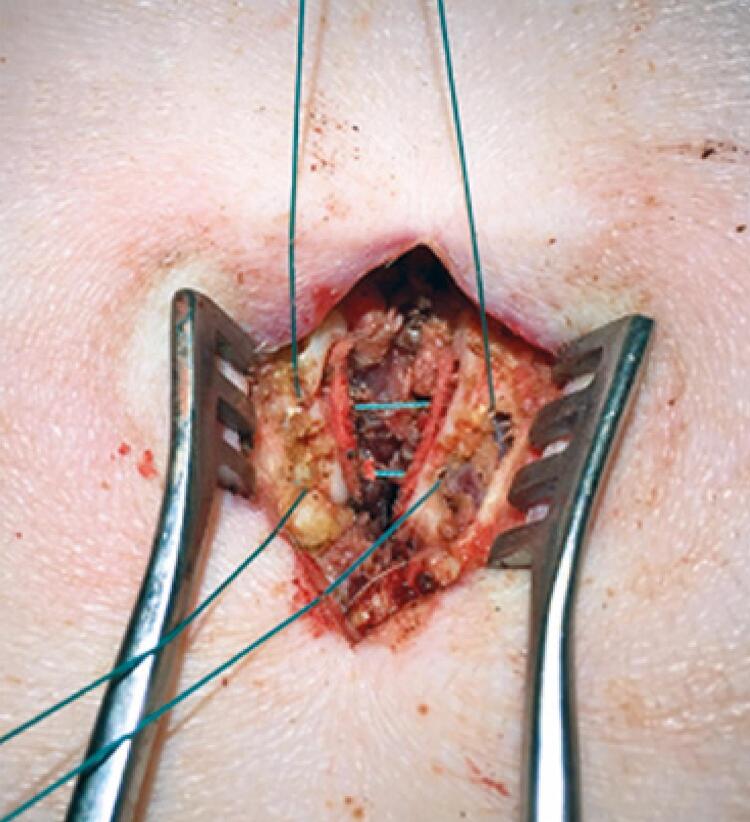
Closing of osteotomy of the spinous process by transosseous sutures

A laminectomy and excision of the ligamentum flavum were performed. Then, the previously osteotomized spinous processes were close with transosseous sutures (Figure 4), followed by sutures of the subcutaneous and skin.

The strength was the transosseous suturing training of the human-like spinous processes. The weak point, on the other hand, was the fact that the spinous process was deeper, making it difficult to perform the osteotomy – especially, in the ventral portion of the spinous process.

### Retropleural miniaccess to the thoracic spine technique

The procedure began with an incision between 3 to 5cm over the level of the vertebra of interest. The rib fragment corresponding to the level of interest was resected retropleurally. Costal resection was performed subperiosteally, avoiding injury to the inferior vascular-nervous bundle and the underlying parietal pleura.

After resection, blunt digital divulsion was performed between the rib and the parietal pleura, up to the head of the rib. The head of the rib led to the intervertebral disc.

The distance between the region of the intervertebral disc and the surface of the skin was measured, and adequate depth blade retractors were placed. Then, the retractor was introduced carefully so as not to injure the parietal pleura and adjacent lung. Anteroposterior distance was acquired according to the blade over the parietal pleural and lung.

Vertebral corpectomy was performed, using drill, chisels and curettes. Bleeding was tamponed with gauze mounted on a long instrument (Mixter), in addition to hemostatic paste or cotton agents.

At the end of training and with the removal of the retractor, lung expansion was able to tamponade the extra pleural space. Additional hemostatics could be placed if required.

Strengths were rib cage anatomy allowing human-like access and the opportunity for spine surgeons to become familiar with the surgical anatomy of the chest. The weakness was only training a few students per animal (only two accesses performed, on different sides).

### Retroperitoneal access to lower lumber spine technique

Synframe soft tissue retractors (Depuy Synthes, US) and fluoroscopy were used for this training session.

A lower transverse abdominal incision was made after fluoroscopic imaging. Alternatively, if the objective was exposing several levels of the lumbar spine, it was possible to start with a longitudinal marking on the skin over the levels of the spine to which access was aimed.

After skin and subcutaneous incision and muscle plane divulsion, the peritoneal membrane, thinner than in humans, was identified. A blunt hand dissection was performed, and generally, a swab was used to move the peritoneal sac away from the abdominal musculature, on the left side of the midline, for the aorta was located on that side. From the right, the vascular structure initially identified in the spine path was the vena cava (as well as the confluence of the iliac veins to the inferior vena cava). This is a more friable structure whose injury is more dangerous.

At the time of the blunt dissection of the retroperitoneal plane, the first complication, the unintentional opening of the peritoneal sac, could occur. When it occurred, an immediate suture was performed so that the peritoneal content would not hinder the progression of the dissection.

The psoas muscle was the boundary of the dissection plane from the peritoneum towards the spine. Cranially, the kidney, a retroperitoneal structure, could be identified. The identification indicated the dissection was taking place at higher levels of the lumbar spine.

There were small branches between the iliac vessels and the spine, which had to be dissected, in addition to a double ligation with cotton thread, in order to separate spine vessels.

The opportunity for spine surgeons to become familiar with retroperitoneal surgical anatomy and the management of complications, such as injury to the peritoneum or large vessels, were the strengths. The weakness was the difficulty in locating the skin incision site anatomically, and therefore fluoroscopy is recommended before starting the procedure.

### Percutaneous pedicular instrumentation technique

Different lumbar implants were used in this technique: Aesculap S4 spinal system Germamy; Viper Prime™ (Depuy Synthes, US) and CD Horizon™ (Medtronic), in addition to fluoroscopy. The swine pedicular instrumentation technique was similar to the one for humans, given the pedicle proportions were comparable to the anteroposterior and upper-inferior diameters. However, due to the fact that the anteroposterior diameter was reduced, the maximum length of the implants used safely was 30mm.

The level of instrumentation was identified through fluoroscopy. The anteroposterior image aims to show the spinous process in the midline and the upper plateau above the pedicles of interest.

A 1-2cm incision was made laterally to the lateral edge of the pedicle and deepened to the dorsolumbar fascia, which was divulged with blunt material or electrocautery.

With the observation of anteroposterior fluoroscopy, a Jamshidi needle was introduced into the bone, with an entry point on the lateral edge of the pedicle and parallel to the disc space. When the needle touched the medial pedicle edge, it switched to the lateral fluoroscopic view.

A Kirschner wire was introduced through a Jamshidi needle, which was removed. The Kirschner wire guided subsequent instrumentation, including the pedicular screw.

**Figure 5:**
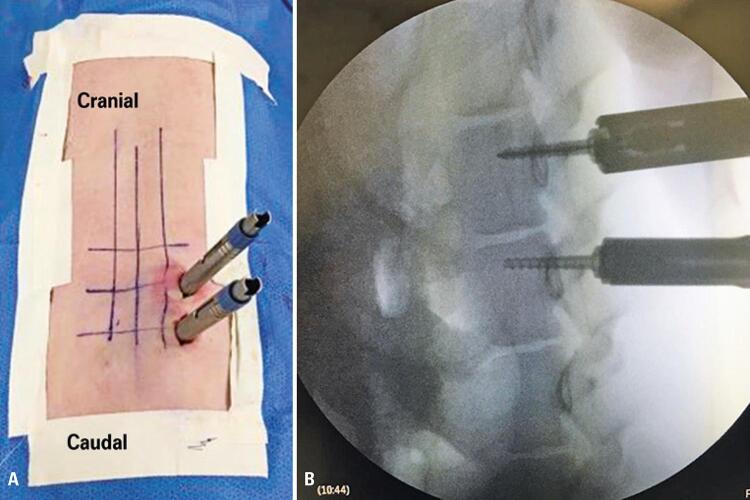
(A) Intraoperative view of dilators positioning for percutaneous pedicular screws; (B) Radioscopy image of percutaneous pedicular screws

A dilator was introduced over the Kirschner wire, followed by a cannulated reamer and introduction of the pedicular screw (Figures 5A and 5B). For each system, there were technical variations, according to characteristics of implants.

The strengths were the execution of a simple and objective exercise; the vertebral pedicle with a large diameter, which facilitates the insertion of screws; and several students practicing on the same animal. The weaknesses were the vertebral body having a narrow anteroposterior diameter, requiring the use of small screws (maximum of 30mm), even in large animals, such as those used in the present study.

### Complication management

The swine model has also been shown to be useful in suture training for dural lacerations. After laminectomy, an intentional laceration was performed on the duramater with a scalpel. The objectives were to train the dura mater suture in the presence of cerebrospinal fluid (CSF) leak and profuse bleeding, in a small space. Students were required to control epidural bleeding with adequate use of hemostatic agents, expand the laminectomy to expose the entire dural lesion, and proceed with the repair of the dural lesion. To perform the exercise, delicate forceps and needle holders were used for suture of the dura, long electrocautery, hemostatic agents in paste, and cotton strips soaked with thrombin.

The possibility of realistic simulation of potentially catastrophic situations, with bleeding and CSF in the surgical field, was a strength. The need for complete infrastructure for its performance (delicate and long surgical instruments, different types of hemostatics, microscope with adequate image definition and long bipolar electrocautery) was a weakness.

## DISCUSSION

Historically, training new surgeons takes place in a “masters and apprentices” system, in which the trainee physician initially observes and subsequently performs surgeries on patients, under the supervision of their hierarchical superiors.^(9)^ However, the increase in bioethic requirements in terms of patient safety,^(10)^ and the development of surgical techniques with longer learning curves^(11)^ have boosted the development of different types of training for surgeons, such as simulations on cadavers, live animals, and software.

The main advantages of using live animals for training surgical techniques are tissue realism, the presence of bleeding, and the possibility of adequately performing hemostasis, in addition to the division of tasks and responsibilities in the operating field.^(12-14)^ In addition, surgical simulation methods help students develop psychomotor and cognitive skills before coming into contact with patients. Surgical simulations can improve the quality of care and operative results of young surgeons.^(15-17)^

Porcine animal models are used in surgical training in several areas of medicine.^(11,18,19)^ There are descriptions of training in laparoscopic abdominal, gynecological, cardiological and plastic surgeries in the literature.^(19-22)^ In addition, the porcine spine has several similarities to humans, which is the main reason why animals are often used for training. Some previous studies compared porcine and human vertebral anatomy.^(11,17)^Based on this knowledge, the authors hypothesized which surgical techniques should be explored as training for spine surgeons.

Regarding anatomy, the porcine pedicular diameter is larger than that of humans.^(11-17)^ In addition, the porcine vertebral body is narrower latero-laterally and antero-superiorly.^(17)^This explains the fact that, in the present study, the pedicle screw insertion was considered feasible and similar to that performed in humans. The length of implants was reduced (maximum of 30mm), and with a 5mm diameter.

On the other hand, the porcine intervertebral disc is rudimentary and narrower than the human one.^(11-24)^ This explains the difficulty in performing some procedures, such as discectomy or placement of cages, regardless of the posterior, lateral or anterior access route. This was the main anatomical limitation found in the animal model under consideration. However, even in techniques such as microsurgical or endoscopic discectomy, training the main steps of procedures was possible, such as retractor positioning, access and exposure route, distance, and neural protection, to develop even more relevant skills than disc excision itself.

Narrow lumbar canal decompression techniques (over-the-top and splitting) of the spinous process were considered similar to those performed in humans. In both scenarios, the main differences pointed out were longer spinous processes and thinner ligamentum flavum in pigs, facts that meant small technical adjustments, such as choice of long retractors. On the other hand, dural sac and root arrangement was similar to humans. It also enabled complication management exercises like bleeding and durotomy to be more real. At that time, there was a pressure on surgeries due to a scenario of complications, a relevant fact for training surgeons, and that is not easily mimicked in other types of training, such as cadavers or computerized simulators.

No measurement of knowledge retention of students or subsequent practical use of new acquired knowledge are considered limitations of this study. In addition, despite the anatomical limitations described for each surgical technique, and the fact that training on animals can be considered costly because it requires veterinary infrastructure, it is considered that, to improve learning curves in such specialized techniques, spine surgeons with experience in animal surgery, in the models presented here, should be part of the training.

## CONCLUSION

The porcine model for simulation of minimally invasive surgical techniques of the spine presented similarities to surgeries performed on humans, and was feasible for training surgeons.
